# Nonmelanoma Skin Cancer in the Heart of the Middle East: Analysis of Mohs Micrographic Surgery Cases From a Tertiary Care Center in Lebanon

**DOI:** 10.1155/jskc/2696706

**Published:** 2024-11-26

**Authors:** Ahmad Berjawi, Namir Saade, Zeina Tannous

**Affiliations:** Department of Dermatology, Gilbert and Rose Marie Chagoury School of Medicine, Lebanese American University, Beirut, Lebanon

## Abstract

Skin cancer is the most common cancer worldwide. According to the Skin Cancer Foundation, Mohs micrographic surgery (MMS) is considered the most effective technique for treating nonmelanoma skin cancer (NMSC). Recurrence rate after MMS for treating NMSC ranges from 1.4% to 3.2% for primary tumors and 2.4%–6% for recurrent tumors. The aim of the study was to report data from a tertiary care center in Lebanon providing MMS to patients with NMSC. Retrospective cohort study was conducted through chart review of 94 patients at the Lebanese American University Medical Center (LAUMC-RH) with a total of 115 cases of MMS identified. The study showed that most cases were males (72; 63%), and 77% were aged > 60 years (88). The average tumor size was 1.6 cm. Recurrence rate was zero in primary tumors and 0.9% in recurrent tumors. With respect to age, bivariate analysis showed that cases of males over 60 years of age were more significantly associated with undergoing Mohs surgery (69% vs. 31%; *p*=0.012). With respect to maximum tumor diameter (MTD) > 1 cm, male gender was associated with a higher MTD when compared to females (74% vs. 26%; *p*=0.02). Also, Area L was associated with a larger MTD when compared to areas H and M, respectively (90% vs. 61.1% vs. 37.7%; *p*=0.01). Multivariate analysis of MTD showed that tumors with MTD > 1 cm were significantly associated with male gender, presence in low- or middle-risk areas and being a recurrent tumor. This study shows that MMS is adequate for the treatment of NMSC at our center with minimal complications (< 1%) and recurrence (< 1%).

## 1. Introduction and Literature Review

Skin cancer remains to be the most common cancer worldwide [1]. Basal Cell Carcinomas (BCC) and Squamous Cell Carcinomas (SCC), termed nonmelanoma skin cancers (NMSC), have an ever-increasing incidence year by year with an approximate increase by 33% between the years 2007 and 2017 [2]. According to The Skin Cancer Foundation, about 3.6 million BCCs and 1.8 million SCCs are diagnosed annually in the United States alone [3]. European data highlights that BCCs have a greater increase in incidence rate than SCCs, although the latter is also steeply increasing [4, 5]. Invasive melanoma, on the other hand, only accounts for < 1% of all skin cancers in the US every year but carries a larger burden of disease, as NMSC generally carry low metastatic potential [1]. 5-year recurrence rate for NMSC treated with surgical excision ranges from 3.2% to 10% in primary BCC and up to 17% in recurrent BCC [6]. Likewise, 5-year recurrent rate for SCC ranged from 5% to 18.7% for primary SCC and up to 23% for recurrent SCC [7]. Mohs Micrographic Surgery (MMS) is a precise form of skin cancer excision done in stages on the same day. An initial excision aims to remove visible tumoral tissue along with a thin margin. Frozen sections are obtained and examined under the microscope for mapping of tumoral extension and margin clearance. In case of margin positivity, the Mohs surgeon will selectively re-excise again and re-examine the sectioned tissue under the microscope. Following tumoral clearance, a reconstruction ensues for defect repair [8]. According to the national Spanish Mohs registry, MMS offers a 5-year cure rate of 98.7% for BCC and 95.5% for SCC [9]. This is primarily achieved by the possibility of examining 100% of the tumor margins with MMS, as opposed to < 1% of tumor margins with standard excisions with predetermined margins [10]. In Australia, the trend in Mohs surgery use for NMSC increased by 415% from 1997 till 2017 [11]. Several risk factors are associated with increased risk of recurrence including preoperative size of tumor, location, and recurrent tumors. Therefore, specific criteria for MMS use were published in 2012 by the American Academy of Dermatology [12]. Data from Brazil on the use of MMS for BCC showed that most (87.1%) BCC were located in the H-zone, 50.5% were primary tumors and 56.4% had large tumor diameter > 2 cm [13]. Moreover, Pugliano-Mauro et al., in his study on cutaneous SCC treated with MMS had an average age of 70.6 years low recurrence rate of 1.3% [14]. 5-year recurrence rate of BCC post MMS treatment from Sweden was 2.1% for primary BCC and 5.2% for recurrent BCC, average age was 69 years, and most common location is area H [15]. Epidemiological studies lack in Lebanon regarding the incidence, risk factors, and MMS use for NMS. Our retrospective cohort study aims to report data from a tertiary care center in Lebanon providing MMS to patients with NMSC. We also attempt to establish statistically significant linkages between different parameters of our MMS cases to better elucidate our understanding of NMSC, and its subsequent treatment in Lebanon.

## 2. Methods

**Figure 1:**
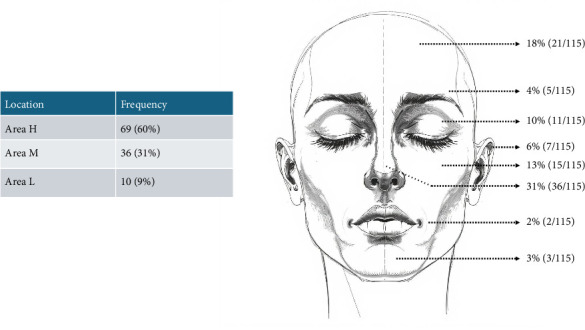
Tumor location according to recurrence risk.

A retrospective cohort study was conducted through reviewing files of 94 patients having undergone MMS for NMSC with a total of 115 MMS cases identified. Every file was given a unique identification number, as initials and names were not to be used in data collection. For every MMS case, data were retrospectively collected for patients' demographics, tumor characteristics, MMS aspects, and complications (parameters collected outlined in Table 1). At our center, the AAD 2012 Mohs surgery criteria were used for patient selection [12]. Data were collected over a 6-month period (December 2022–June 2023), and files reviewed were over 10 years period ranging from 2013 to 2023. Tumors were classified as being located on either areas H, M, or L: H pertaining to areas of high-risk of recurrence such as the mask areas of the face, eyebrows, nose, lips, genitalia, hands, feet, nail units, ankles, and nipples. Area M pertained to areas of moderate risk of recurrence such as the cheeks, the forehead, the scalp, the neck, the jawline, and pretibial surfaces. Finally, area L pertaining the lowest risk of recurrence and includes the rest of the body (i.e., trunk and extremities).  Figure 1 outlines a visual summary of the above-mentioned areas (H, M, and L) [8, 13].

### 2.1. Statistical Analyses

All statistical analyses were conducted using the Statistical Package for the Social Sciences software (SPSS) version 28.0. Categorical variables were explored and summarized using frequency and percentage. The summary of the continuous variables was reported using the mean, standard deviation, and confidence intervals. Chi-square test was used to test association between patients and tumor characteristics when more than 2 variables are assessed, and Fisher's exact test was used to associate patients and tumor characteristics with NMSC when 2 variables were assessed (using 2 × 2 table).

Simple and multiple logistic regression models were used. Variables with a *p* value less than 0.05 on bivariate analysis were used for the logistic regression models.

### 2.2. Statement of Ethics

This study was approved by the LAU Institutional Review Board (reference #: LAUMCRH.ZT1.16/Mar/2022) and conducted according to their standards, applicable government regulations, and institutional research policies and procedures.

## 3. Results

### 3.1. Demographic Data

Demographic data pertaining to our sample are summarized in Table 1. A total number of 115 cases were reviewed. Most of the cases were males (72; 63%), and 77% were aged > 60 years (88). 74 cases (64%) had their lesion present for over 1 year. The cases that were referred from private clinics totaled 54 patients (47%).

### 3.2. Tumor Characteristics

The average tumor size was 1.6 cm, and the maximum tumor diameter (MTD) among the cases was > 1 cm in 50% of the cases (*n* = 57). The most common location was area H (69; 60%) followed by area M (31%) (Figure 1). Concerning the histological subtype of the tumor, BCC was more common than SCC (80% vs. 20%). Low-risk BCC (nodular or superficial subtype) and high-risk BCC (infiltrative, morpheaform, micronodular) were equal among the cases (41 vs. 41). 10 cases were recurrent tumors compared to 105 primary tumors (9% vs. 91%).

### 3.3. Surgical Data

Most cases needed 1 or 2 stages of Mohs surgery (23 and 64 cases respectively; 76%), and 28 cases (24%) needed 3 or more stages of Mohs surgery. Graft or flap repair was needed in 70 cases compared to 45 primary complex linear closure or secondary intention healing cases (61% vs. 39%). To note that 5 cases needed plastic surgery referral for repair with either a graft or a flap. Duration of surgery was 6 h or more in 38 cases (33%). One patient necessitated enucleation by ophthalmology following failure to clear the tumor via MMS. Additionally, 1 MMS was done for dermatofibrosarcoma protuberans, and another slow MMS was performed for a patient with a lentigo maligna melanoma.

### 3.4. Complications or Recurrence

No case of uncontrolled haemorrhage was reported. Surgical wound infection was reported in 1 case only (< 1%) and recurrence rate was < 1% (1/115 cases).

### 3.5. Bivariate Analysis

With respect to age, bivariate analysis showed that cases of males over 60 years of age were more significantly associated with undergoing Mohs surgery (69% vs. 31%; *p*=0.012) (Table 2). With respect to MTD > 1 cm, male gender was associated with a higher MTD when compared to females (74% vs. 26%; *p*=0.02). Also, area L was associated with a larger MTD when compared to area H and M: 90% of tumors in area L area were > 1 cm in diameter compared to 38% of those in area H and 61% in area M (*p*=0.01) (Table 3). Ninety percent (90%) of recurrent tumors had a MTD > 1 cm in comparison to 45% of primary tumors (*p*=0.008). Coincidentally, all 6 cases of SCC in situ were > 1 cm (*p*=0.018). Multivariate analysis of MTD showed that tumors with MTD > 1 cm were significantly associated with male gender (*β* = 3.0, 95% CI [1.1–8.1], *p*=0.028) presence in area L or M (*β* = 4.3, 95% CI [1.4–12.6], *p*=0.007) and being a recurrent tumor (*β* = 16.1, 95% CI [1.6–157.7], *p*=0.017). [*β* refer to beta coefficient on multivariate analysis, 95% CI refers to 95% confidence interval on multivariate analysis].

## 4. Discussion

NMSC incidence is increasing worldwide, justifying the need for assessment of treatment modalities that are being evaluated [16, 17]. Mohs surgery remains the gold standard for NMSC with specific indications according to the American Academy of Dermatology [12]. The use of Mohs surgery for NMSC increased by more than 400% in the last 20 years [11]. This study evaluated Mohs surgery use for NMSC in a single center in Lebanon. 115 cases were reviewed and showed that males more commonly underwent MMS for NMSC than females did (63%). This data mirrors previously available data in the medical literature, which also reflected that males more commonly were subjects of MMS than females (59% v 41%) [18]. A larger proportion of our patients were above the age of 60 compared to what has been reported in the national Mohs Data trend (77% vs. 65%) [18]. 64% of the cases had the lesion for more than a year which highlights the late presentation for NMSC in our population.

The average tumor size was 1.6 cm^2^, significantly less than the average size reported in the literature which was reported by Barbieri et al. as 3.3 cm^2^ [19]. Additionally, most tumors fell within area H (60%), the second most common location was area M (31%). The literature, however, reports a higher number of tumors falling within area H (75%–88%) [13, 20]. BCCs were more common than SCCs in our cohort (80% vs. 20%), which is similar to worldwide NMSC incidence [21]. However, trend of MMS for SCC is increasing in Australia, as reported by Stewart et al. where the incidence of SCC increased from 6% in 1997 to 11% in 2017 [11]. Interestingly, our study revealed that high-risk BCC and low-risk BCC have similar incidence (50% vs. 50%), as opposed to data in literature which portrayed a higher incidence of high-risk BCC (69% vs. 31%) [13]. While the literature on the use of MMS for the removal of BCC and SCC dealt prominently with recurrent tumors (57%–62% primary; 38%–43% recurrent), our cohort showcased most tumors being primary (90% primary vs. 10% recurrent).

Three or more stages of MMS were needed in 24% of the cases which is a higher percentage than previously reported by Fantini et al. on BCC (11%) [13]. Moreover, tissue repair was primarily achieved by the means of grafts or flaps (61%) compared to 39% of the cases who underwent either primary linear complex closure or secondary intention healing. Similarly, in their study on evolution of MMS in Australia, Stewart et al. reported an increase in primary linear complex closure or secondary intention healing from 16% in 1997 to 42% in 2017 [11].

Surgical complications in the study were minimal, no uncontrolled haemorrhage was reported and 1 patient (< 1%) suffered from a surgical wound infection that was successfully treated with oral antibiotics. The data is consistent with data in the United States on adverse events of MMS (< 1%) reported by Alam et al. [22] Additionally, their study on 20,821 MMS cases showed that surgical wound infection is the most common adverse event reported (61% of all adverse events) [22]. Recurrence rate in our study was 1%, lower than what was reported in the literature, despite the most common location of the tumors excised being in area H which holds a high risk for recurrence (60%). While following up the cohort for a maximum period of 10 years, we report only one case of recurrence in an 85-year-old male who initially presented for a recurrent SCC of the shoulder. However, data from Australia on MMS for BCC and SCC showed a recurrence rate of 1.6% for primary BCC and 2.6% for primary SCC over the span of 5 years of follow up [6, 7].

Males above 60 years of age were 4 times more likely to undergo MMS in this study (OR = 4.5; CI = 1.6–12.5). Also, male gender was associated with a MTD > 1 cm (OR = 3; CI = 1.1–8.1). This may be in part due to the higher incidence of MMS in males in our study, similar to the national data [18]. Area L and M were more likely to be associated with a MTD > 1 cm compared to area H (*p* < 0.05), a finding that goes against previously reported data by Fantini et al. on BCC where 72% of tumors > 1 cm in MTD were located on area H [13]. Recurrent tumors were more likely to have an MTD > 1 cm (90% recurrent vs. 45% of primary). This is similar to data on BCC and SCC in Australia, noting that 37% of primary BCC and 25% of primary SCC had a tumor size < 1 cm [7, 23].

### 4.1. Limitations and Conclusion

The main limitation of the study was the small sample size which may not be representative of the whole Lebanese population. Another limitation is the study being a retrospective cohort study where a 5-year follow up may not be documented in all patients or in those who have been lost to follow up appropriately. Finally, the lack of a registry in Lebanon on skin cancer in general, and NSMC specifically, as well as the scarcity of medical centers offering MMS in Lebanon, restrains our ability to draw nationwide comparisons between our center and the rest of the country. Thus, making our study a pilot study in Lebanon.

NMSC remains to rank as the most prevalent cancer worldwide, and MMS is the gold standard for treatment when specific criteria are met. MMS in Lebanon is on the rise and few centers provide this mean of treatment. This study shows that Mohs surgery is essential for treating NMSC in our institution, with minimal recurrence rate and complications. MMS done for BCC is higher than SCC in this cohort. Factors associated with MMS use are age > 60 years, and male gender. Larger tumor diameter was found in male gender, location L and M, and recurrent tumors. This is the first study in Lebanon to assess the use of MMS for NMSC, and the patient's characteristics associated with its use. Building upon our retrospective cohort study on MMS for NMSC, further work would be focused on longer follow-up periods to further assess recurrence. Comparing treatment outcomes between MMS and standard surgical excision, as well as cosmetic outcomes between MMS repair and standard surgical excision would also be intriguing scopes to investigate.

## Figures and Tables

**Table 1:** Patients demographics and tumor characteristics.

Characteristics	Average
Age	68 years
Sex	1.75: 1 ratio (72M vs. 43F)
Location	91% on face versus 9% on body
Types	80% BCC, 20% SCC
Average size preop	1.6 **cm**^2^
Wound infection rate	**1/115 = 0.9%**
Recurrence rate	**1/115 = 0.9%**
Average time of surgery	4.8 h
Average number of stages	2.2 stages

*Note:* The bold values signify the low wound infection rate and recurrence rate. Also, the average size preop which is more than 1 cm squared.

**Table 2:** Bivariate analysis comparing gender to age.

	Age	
	< 60 years (%)	> 60 years (%)
Sex		
Female	16 (59.3%)	**27 (30.7%)**
Male	11 (40.7%)	**61 (69.3%)**

*Note:* Fisher's exact test used *p*=0.012. The bold values signify the significant difference in male individuals > 60 years of age having MMS compared to females.

**Table 3:** Bivariate analysis comparing location to MTD.

	Maximum tumor diameter (MTD)	
	< 1 cm (%)	> 1 cm (%)
Location		
Area H	43 (62.3%)	**26 (37.7%)**
Area M	14 (38.9%)	**22 (61.1%)**
Area L	1 (10%)	**9 (90%)**

*Note:* Chi-square test used *p*=0.012. The bold values signify the significance of having a maximum tumor diameter > 1 cm being common in areas M and L.

## Data Availability

The data that support the findings of this study are available from the corresponding author upon reasonable request.
